# Tulane University

**Published:** 1889-07

**Authors:** 


THE TULANE UNIVERSITY MEDICAL DEPARTMENT has an advertisement in this Journal to which we invite attention.

